# Epidemiology of tobacco use and nicotine dependence in truck drivers

**DOI:** 10.11606/s1518-8787.2022056003698

**Published:** 2022-11-18

**Authors:** Inaina Lara Fernandes, Rafael Alves Guimarães, Roselma Lucchese, Ivânia Vera, Rodolfo Pereira de Brito, Camila Borges Ramos, Tamíris Augusto Marinho, Patrícia Silva Nunes, Henrique Senna Diniz-Pinto, Thiago Aquino Amorim

**Affiliations:** I Universidade Federal de Catalão Instituto de Biotecnologia Catalão Goiás Brasil Universidade Federal de Catalão. Instituto de Biotecnologia. Catalão, Goiás, Brasil; II Universidade Federal de Goiás Faculdade de Enfermagem Goiânia Goiás Brasil Universidade Federal de Goiás. Faculdade de Enfermagem. Goiânia, Goiás, Brasil; III Universidade Federal de Goiás Instituto de Patologia Tropical e Saúde Pública Goiânia Goiás Brasil Universidade Federal de Goiás. Instituto de Patologia Tropical e Saúde Pública. Goiânia, Goiás, Brasil; IV Instituto Federal de Educação, Ciência e Tecnologia de Goiás Goiânia Goiás Brasil Instituto Federal de Educação, Ciência e Tecnologia de Goiás. Goiânia, Goiás, Brasil

**Keywords:** Tobacco Use Disorder, epidemiology, Nicotine, Motor Vehicles, Occupational Health, Brazil

## Abstract

**OBJECTIVE:**

To investigate the epidemiology of tobacco use and nicotine dependence in a sample of truck drivers in Brazil.

**METHODS:**

Between 2015 and 2016, a cross-sectional study was conducted on 624 truck drivers who operate on the BR-050 highway in Brazil. Participants were interviewed about sociodemographic data, occupational characteristics, mental health, behavioral data, and tobacco use. Then, the Fagerstrom test for nicotine dependence (FTND) was used to verify nicotine dependence in smoking truck drivers. Logistic regression and linear regression were also used to verify factors associated with tobacco use in the previous 30 days and nicotine dependence scores, respectively.

**RESULTS:**

The prevalence of tobacco use among truck drivers was 21.1% (n = 132;95%CI: 18.1–24.5). Of the total number of smokers who responded to the FTND (n = 118; 89.4%), most had high/very high nicotinic dependence (68.6%; 95%CI: 59.8–76.3). Tobacco use was associated with absence of religion (adjusted odds ratio [AOR]: 2.60; 95%CI: 1.35–5.01), employment relationship of the contract (AOR = 1.98; 95%CI: 1.26–3.13); > 12 hours daily working time (AOR = 1.80; 95%CI: 1.09–2.98) and alcohol use in the previous 30 days (AOR = 2.92; 95%CI: 1.86–4.57). Irregular physical activity was associated with higher scores of nicotine dependence (β = 1.87; 95%CI: 0.55–3.19).

**CONCLUSION:**

The results showed a high prevalence of tobacco use and high/very high nicotine dependence among the truck drivers.

## INTRODUCTION

Tobacco use and nicotine dependence represent serious public health problems. The use of nicotine highly impacts morbidity and mortality, mainly due to the increased risk of developing chronic non-communicable diseases (NCDs). An estimated 1.3 billion people use tobacco worldwide; in addition, the World Health Organization estimates that the use of this substance accounts for 7 million deaths annually^1^. In countries in the Americas, such as Brazil and the United States (US), the prevalence of smoking has been declining in recent years, due to public smoking-control policies; however, it remains high^2^.

Truck drivers constitute a group highly vulnerable to tobacco use and nicotine dependence. This population possess a unique lifestyle, often with long daily working hours^3,4^; easy access to licit and illicit substances; experiencing occupational stress^5,6^, fatigue, increased risk of traffic violence, and various assaults^7,8^; and adopting less healthy lifestyles. These conditions increase their risk of tobacco use, as reported in the literature.

In Brazil, few studies have been conducted on truck drivers’ tobacco use^3,4,9^ and, to our knowledge, none have investigated the use of this substance and nicotine dependence as dependent variables and sought to identify their associated factors. Such data are key for planning multifaceted actions and public policies to control the use of this substance in this population, such as permanent education for health care teams, especially primary health care for screening and smoking cessation interventions in this population; expansion of the treatment of nicotine dependence in the Unified Health System (SUS), including non-drug and drug aspects; specific campaigns for health education aimed at truck drivers, strengthening worker health promotion strategies in transport companies; periodic adjustments to the main taxes on cigarettes and the prices of these products at retail; promotion of tobacco-free environments, including those roadside establishments; and strengthening of tobacco control policies in Brazil, like the National Anti-Smoking Program and cross-cutting policies such as the National Health Promotion Policy and the National Primary Care Policy, among others. Thus, the objective of this study is to investigate the epidemiology of tobacco use and nicotine dependence in a sample of truck drivers in Brazil.

## METHODS

This is a cross-sectional study that was conducted among truck drivers traveling on the BR-050 highway, which connects the cities of Catalão (Goiás State), Araguari and Uberlândia (Minas Gerais State). The BR-050 is a federal radial highway, beginning in the capital city, Brasília (Federal District), and ending in the Santos city (São Paulo State); it crosses the states of Goiás, Minas Gerais, and São Paulo.

In this study, inclusion criteria were: (i) having been a truck driver for at least 30 days, and (ii) having traveled on BR-050.

To determine the minimum sample size required, we set a significance level of 95.0% (α = 0.05), and the prevalence of tobacco use in Brazilian truck drivers of 22.2%^10^; then, a 30.0% rejection rate was added to this value. Consequently, we found a minimum sample requirement of 346 drivers. However, to increase the power of the sample to detect associations especially of variables with few observations we collected more than twice the minimum sample. The sampling used in the study was non-probabilistic for convenience.

The participants were recruited from five rest or recharge/discharge courtyards near the BR-050; specifically, three of these locations were in the city of Catalão (state of Goiás), and one each in Araguari and Uberlândia, both in the state of Minas Gerais. These points are strategic locations where truck drivers can eat, wash/use restrooms, and load/unload products. Further, these data-collection sites reflect the economic characteristics of the region, which are mainly related to the automobile industry and the manufacturing of agricultural machinery, but also include mining, production of feed, and creation of implements used in crop farming. The transport flow through this locality constitutes part of the distribution process of products to many Brazilian states. Note that one of the largest transport companies in Latin America is based in this region; moreover, the cities of Catalão, Araguari, and Uberlândia also have grain and fertilizer loading and unloading companies, as well as benefited products that are exported to ports by large rural producers.

Data collection was conducted between May 2015 and January 2016 during business hours (daytime) or after the commercial working day (night), including while the drivers were at rest stops, staying in overnight accommodation, having meals, and during loading/unloading of products. All drivers, after parking their vehicles, were individually approached and invited to participate in the study. They were then given information on the purpose, methods, benefits, potential risks of the study, and were also informed that their participation was purely voluntary and that their responses would be confidential. Those who consented were interviewed, face to face, by trained field researchers. Each interview lasted approximately 30 minutes.

A structured questionnaire was used and focused on sociodemographic data, labor characteristics, tobacco use, and other potential associated factors. Nicotinic dependence was verified with the Fagerstrom test for nicotine dependence (FTND), an instrument consisting of six items, and which is easy to understand and apply; further, it was validated in Brazil in 2002^11^. The scores obtained from the test facilitate classifying nicotine dependence into five levels: very low (zero to two points); low (three to four points); average (five points); high (six to seven points); and very high (eight to 10 points)^11^. Regarding degree of dependence, using the FTND scores addiction severity can be grouped into very low/low (category one), medium (category two), and high/very high (category three).

Dependent variables were set as: (I) tobacco use in the previous 30 days, collected with the question “Have you smoked (cigarettes, tobacco product) at least once in the last 30 days?”, dichotomized into 0 “No” or “Yes” and (II) nicotine dependence scores. The independent variables included in the analysis were:

(I) Sociodemographic: age group (years), calculated from the difference between the date of birth and the date of the interview – age groups were categorized every 10 years up to 50 years or older: 21–29, 30–39, 40–49 or ≥ 50 years; marital status (married, single, or separated/divorced/widower), collected with the question “What is your current marital status?”; race/skin color, categorized as white, mixed race (pardo), black, and others (Asian or Native American), due to the small number of observations – the classification was carried out as established by the *Instituto Brasileiro de Geografia e Estatística* (Brazilian Institute of Geography and Statistics)^12^; religion (yes or no), collected with the question “What is your religion?”, given options were “evangelical”, “catholic”, “others” or “none”; the option “none” was considered as no and the others as yes for this variable; has children (no or yes), collected with the question “Do you have children?”; monthly income (BRL), collected with the question “What is your monthly household income (in BRL)?”, and categorized into BRL ≤ 2,500 (up to the 25th percentile), 2,501–3,000 (up to the median) or > 3,000 (greater than median) and schooling group (years), measured by the question “Up to what grade did you study?”; this variable is calculated in complete years from the participant’s response of the school grade. For example, if the participant answered “first grade of elementary school” it was considered a complete year of formal study. This variable was categorized as ≤ 4, 5–8 or > 8 years.

(II) Laboral: employment relationship (self-employed or under an employment contract), collected with the question “What is your current employment relationship?”; we defined freelance truck drivers (self-employed) as truck owners who work on their own or provide an outsourced service to companies, and trucker employees as those with a permanent link to a company^13^; experience as a truck driver group (years), collected with the question “How long have you been working as a truck driver (in years)?” and categorized into ≤ 9 (up to the 25th percentile), 10–17 (up to the median) or > 17 (greater than median); daily working (hours), collected with the question “How many hours do you travel per day on average?” and categorized into ≤ 10 (up to the 25th percentile), 11–12 (up to the median) or > 12 (greater than median).

(III) Mental health: self-reported anxiety (no or yes), collected with the question “Have you ever been diagnosed with anxiety by a doctor?”; self-reported depression (no or yes) collected with the question “Have you ever been diagnosed with depression by a doctor?”; suicidal ideation in lifetime (no or yes), collected with the question “Have you ever thought of taking your own life?”.

(IV) Behavioral: regular physical activity (yes or no) – defined as physical activity of at least 30 minutes on five or more days of the week^14^ and collected with the question “Do you practice physical activity for at least 30 minutes a day five or more times a week?”; alcohol use in the previous 30 days (no or yes) collected with the question “Have you consumed any alcoholic beverages at least once in the last 30 days?”.

### Statistical Analysis

The data were analyzed using Stata software, version 16.0. First, the normality of the quantitative variables was verified using the Kolmogorov-Smirnov test with Lilliefors correction. Next, in the descriptive analysis, the qualitative variables were expressed as absolute and relative frequency, and the quantitative variables as median and interquartile range (IQR) due to the non-normal distribution of these variables.

To verify the factors associated with tobacco use, bivariate and multivariable logistic regression analyses were performed. In the bivariate analysis, each independent variable was associated, one at a time, with the dependent variable. Next, to control for potential confounders, all variables, regardless of statistical significance in the bivariate analysis, were included in a conditional forward method logistic regression model. Additionally, the Hosmer–Lemeshow test, receiver operating characteristic curve (ROC curve), and R^2^ were used to evaluate the quality of the model.

To analyze the factors associated with nicotinic dependence scores, bivariate and multivariable analyses were conducted. In bivariate analysis, Mann-Whitney or Kruskal-Wallis (followed by Dunn’s test in case of statistical significance) were performed to compare median scores between the independent variables. Next, all variables were included in a multiple linear regression model. The model was analyzed for the presence of multicollinearity by the variance inflation factor (VIF); linearity by the graph in the augmented component-plus-residual plot; normality of the residuals by the analysis of the graph and standardized normal probability plot; specification errors by Ramsey RESET test and heterocedasticity by Breusch–Pagan test. R^2^ was presented to verify the explanatory power of the model. In all analyses, p-values of less than 0.05 were considered statistically significant.

### Ethical Aspects

This study was approved by the Human Research Ethics Committee of the Federal University of Goiás, protocol number 1.410.628/2015. All participants provided written consent.

## RESULTS

Of the total number of truck drivers invited to participate in the study (n = 700), 66 refused and 10 had less than 30 days of professional experience. Thus, the final sample consisted of 624 individuals (response rate of 89.1%). The regions of residence (origin) of the participants were: Southeast (n = 276; 44.4%), Central-West (n = 257; 41.4%), South (n = 73; 11.8%), North (n = 8; 1.3%) and Northeast (n = 8; 1.3%).

The median age was 42.0 years ( IQR=35.0−51.0 ); 73.9% were married or had a stable union; 4.78 self-declared white race/color; 92.3% had a religion and 87.0% had children; median monthly household income was BRL 3,000 ( IQR=2,500−4,000 ), and schooling was 8 years ( IQR=5.0−11.0 ). Regarding occupational characteristics, 64.1% had a permanent employment contract; the median experience as truck driver was 17 years ( IQR=9.0−27.0 ) and the daily working time was 12 hours ( IQR=10−14 ). The prevalence of anxiety, depression, and suicidal ideation was 13.3%, 4.0%, and 7.4%, respectively. Finally, 74.5% did not practice regular physical activity and 57.9% used alcohol in the last 30 days (Table 1).

**Table 1:** Characteristics of truck drivers in the sample, 2015–2016.

Variables		
**Sociodemographic**		
Age (years), median (IQR)	42.0 (35.0–51.0)	
Age group (years), n (%)		
18–29	68	10.9
30–39	187	30.0
40–49	195	31.3
≥ 50	174	27.9
Marital status, n (%)		
Married/stable union	461	73.9
Single	110	17.6
Separated/divorced/widower	53	8.5
Race/skin color (self-report), n (%)		
White	298	47.8
Mixed race	236	37.8
Black	68	10.9
Others (Asian or Native American)	22	3.5
Religion, n (%)		
Catholic	436	69.9
Evangelical	118	18.9
Others	22	3.5
None	48	7.7
Active practice, n (%)		
Yes	576	92.3
No	48	7.7
Has children^a^, n (%)		
No	81	13.0
Yes	540	87.0
Monthly income (BRL), median (IQR)	3,000 (2,500–4,000)	
Monthly income (BRL) group, n (%)		
≤ 2,500	169	27.1
2,501–3,000	157	25.2
> 3,000	298	24.2
Schooling (years), median (IQR)	8.0 (5.0–11.0)	
Schooling group (years)		
≤ 4	94	15.1
5–8	273	43.8
> 8	257	41.2
**Occupational**		
Employment relationship, n (%)		
Self–employed	224	35.9
Contract	400	64.1
Experience as a truck driver (years), median (IQR)	17.0 (9.0–27.0)	
Experience as a truck driver (years) group, n (%)		
≤ 9	165	26.4
10–17	157	25.3
> 17	302	48.4
Daily working time (hours), median (IQR)	12 (10–14)	
Daily working time (hours) group, n (%)		
≤ 10	245	39.3
11–12	202	32.4
> 12	177	28.4
**Mental health**		
Anxiety (self–report), n (%)		
No	541	86.7
Yes	83	13.3
Depression (self–report), n (%)		
No	599	96.0
Yes	25	4.0
Suicidal ideation (lifetime), n (%)		
No	578	92.6
Yes	46	7.4
**Behavioral**		
Regular physical activity^b^, n (%)		
Yes	159	25.5
No	464	74.5
Alcohol use (previous 30 days), n (%)		
No	263	42.1
Yes	361	57.9

IQR = interquartile range.

^a^ Missing data: 2.

^b^ Missing data: 1.

The prevalence of the tobacco use in the previous 30 days was 21.1% (95%CI: 18.1–24.5; n = 132). Of the total number of smokers who responded to the FTND (n = 118; 89.4%), most had high or very high nicotinic dependence (68.6%; 95%CI: 59.8–76.3); 18.6% (95.0%CI: 12.6–26.6) low/very low dependence; and 12.7% (95%CI: 7.8–19.9) average dependence. The median FTND score of these individuals was 7 ( IQR=5.0−9.0 ). Additionally, the smokers’ median age of beginning the smoking habit was of 16.0 years (  IQR =15.0−18.0).

The bivariate analysis of the potential factors associated with tobacco use showed association with the following variables: religion, employment relationship, daily working time, regular physical activity, and alcohol use in the previous 30 days (p-value < 0.05) (Table 2). All variables were included in a multiple logistic regression model for adjusted potential confounders. The outcome was independently associated with absence of a religion (adjusted odds ratio [AOR] = 2.60; 95%CI: 1.35–5.01), employment relationship of the contract (AOR = 1.98; 95%CI: 1.26–3.13); > 12 hours daily working time (AOR = 1.80; 95%CI: 1.09–2.98) , and alcohol use in the previous 30 days (AOR = 2.92; 95%CI: 1.86–4.57) . The Hosmer–Lemeshow test showed that the model had a good quality of fit (*x*^2^: 4.41; p = 0.818). Additionally, the area on the ROC curve of the final model was 0.707, indicating an acceptable degree of discrimination between smokers and nonsmokers (Table 3).

**Table 2:** Bivariate analysis of potential factors associated with tobacco use in truck drivers, 2015–2016.

Variables	Total	Tobacco use			OR (95%CI)	p

		n	%	95%CI		
Age group (years)						
18–29	68	17	25.0	16.2–36.4	1.00	
30–39	187	41	21.9	16.6–28.4	0.84 (0.44–1.61)	0.605
40–49	195	44	22.6	17.3–28.9	0.87 (0.46–1.66)	0.682
≥ 50	174	30	17.0	12.4–23.6	0.63 (0.32–1.23)	0.173
Marital status						
Married/stable union	461	95	20.6	17.2–24.5	1.00	
Single	110	27	24.5	17.5–33.4	1.25 (0.76–2.04)	0.366
Separated/divorced/widower	53	10	18.9	10.6–31.4	0.89 (0.43–1.84)	0.896
Race/skin color (self–report)						
White	298	58	19.5	25.4–24.3	1.00	
Mixed race	236	51	21.6	16.8–27.3	1.14 (0.75–1.74)	0.541
Black	68	17	25.0	16.2–36.4	1.37 (0.74–2.56)	0.309
Others (Asian or Native American)	22	6	27.3	13.2–48.2	1.55 (0.58–4.13)	0.380
Has a religion						
Yes	576	114	19.8	16.7–23.4	1.00	
No	48	18	37.5	25.2–51.6	2.43 (1.30–4.51)	0.005
Has children						
No	81	15	18.5	8.0–20.2	1.00	
Yes	540	116	21.5	18.2–25.1	1.20 (0.66–2.19)	0.542
Monthly income (BRL)						
≤ 2,500	169	36	21.3	15.8–28.1	1.00	
2,501–3,000	157	32	20.4	14.8–27.4	0.95 (0.55–1.62)	0.838
> 3,000	298	64	21.5	17.2–26.5	1.01 (0.54–1.60)	0.965
Schooling group (years)						
≤ 4	94	16	17.0	10.7–25.9	1.00	
5–8	273	67	24.5	19.8–30.0	1.58 (0.87–2.90)	0.135
> 8	257	38	19.1	11.0–19.6	1.15 (0.62–2.14)	0.662
Employment relationship						
Self–employed	224	33	14.7	10.7–20.0	1.00	
Contract	400	99	24.8	20.8–29.2	1.90 (1.23–2.94)	0.003
Experience as a truck driver (years)						
≤ 9	165	34	20.6	15.1–27.4	1.00	
10–17	157	33	21.0	15.4–28.1	1.65 (1.03–2.66)	0.039
> 17	302	65	21.5	17.3–26.5	1.97 (1.22–3.19)	0.006
Daily working time (hours)						
≤ 10	245	38	15.5	11.5–20.6	1.00	
11–12	202	47	23.3	18.0–29.6	1.65 (1.03–2.66)	0.039
> 12	177	47	26.6	20.6–33.5	1.97 (1.22–3.18)	0.006
Anxiety (self–report)						
No	541	114	21.1	17.9–24.7	1.00	
Yes	83	18	21.7	14.2–31.7	1.03 (0.59–1.81)	0.898
Depression (self–report)						
No	599	128	21.4	18.3–24.8	1.00	
Yes	25	4	16.0	6.4–34.7	0.70 (0.23–2.08)	0.522
Suicidal ideation (lifetime)						
No	578	120	20.8	17.7–24.3	1.00	
Yes	46	12	26.1	15.6–40.3	1.35 (0.68–2.68)	0.396
Regular physical activity						
Yes	159	22	13.8	9.3–30.1	1.00	
No	464	110	23.7	20.1–27.8	1.71 (1.12–2.61)	0.009
Alcohol use (previous 30 days)						
No	263	34	12.9	6.8–12.8	1.00	
Yes	361	98	27.1	22.8–32.0	2.50 (1.64–3.85)	< 0.001

95%CI: 95% confidence interval; OR: odds ratio.

**Table 3:** Multiple regression of factors associated with tobacco use in truck drivers, 2015–2016.

Variables	AOR	95%CI	p
Has a religion			
Yes	1.00		
No	2.60	1.35–5.01	0.004
Employment relationship			
Self-employed	1.00		
Contract	1.98	1.26–3.13	0.003
Daily working time (hours)			
≤ 10	1.00		
11–12	1.69	0.96–2.60	0.075
> 12	1.80	1.09–2.98	0.021
Alcohol use (previous 30 days)			
No	1.00		
Yes	2.92	1.86–4.57	< 0.001

95%CI: 95% confidence interval; AOR: adjusted odds ratio.

Note: Hosmer-Lemeshow test: *X*^2^: 4.41, p = 0.818; Nagelkerke’s R^2^: 0.123; Area under ROC curve: 0.707.

The bivariate analysis of the median scores and IQR in the FNTD among smokers according to independent variables showed statistically higher nicotine dependence scores in mixed race (*pardo*) individuals when compared with blacks (p-value = 0.027). Dependence scores were also higher in individuals who did not regularly practice physical activity when compared with those who had this practice (p-value = 0.002) (Table 4).

**Table 4:** Bivariate analysis of factors associated with nicotinic dependence scores in truck drivers, 2015–2016 (n = 118).

Variables	FTND score		p

	Median	IQR	
Age group (years)			
18–29	8	5.0–9.0	0.295^a^
30–39	7	5.9–8.0	
40–49	7	4.5–9.0	
≥ 50	8	5.0–8.8	
Marital status			
Married/stable union	7.0	5.0–9.0	0.324^a^
Single	6.0	4.3–7.8	
Separated/divorced/widower	7.5	5.0–9.0	
Race/skin color (self–report)			
White	7.0	5.0–8.0	0.017^a^
Mixed race	8.0	6.0–9.0^c^	
Black	6.0	3.0–7.8	
Others (Asian or Native American)	5.5	2.8–7.5	
Has a religion			
Yes	7.0	5.0–9.0	0.518^b^
No	7.0	4.5–8.0	
Has children			
No	7.0	5.0–8.0	0.885^b^
Yes	7.0	5.0–8.8	
Monthly income (BRL)			
≤ 2,500	7.0	5.0–8.0	0.739^a^
2,501–3,000	7.0	5.0–8.0	
> 3,000	7.0	5.0–9.0	
Schooling group (years)			
≤ 4	7.0	5.5–8.5	0.980^a^
5–8	7.0	5.0–9.0	
> 8	7.0	5.0–9.0	
Employment relationship			
Contract	7.0	5.0–8.0	0.755^b^
Self–employed	7.0	5.0–9.0	
Experience as a truck driver (years)			
≤ 9	7.0	5.0–8.0	0.056^a^
10–17	6.0	4.0–7.0	
> 17	7.5	5.0–9.0	
Daily working time (hours)			
≤ 10	7.0	5.0–8.0	0.075^a^
11–12	6.5	4.3–8.0	
> 12	8.0	6.0–9.0	
Anxiety (self–report)			
No	7.0	5.0–9.0	0.896^b^
Yes	6.0	5.0–9.0	
Depression (self–report)			
No	7.0	5.0–9.0	0.982^b^
Yes	6.5	5.0–8.8	
Suicidal ideation (lifetime)			
No	7.0	5.0–9.0	0.565^b^
Yes	7.0	5.0–8.0	
Regular physical activity			
Yes	5.0	2.8–7.0	0.002^b^
No	7.0	5.0–9.0	
Alcohol use (previous 30 days)			
No	7.0	5.0–9.0	0.828
Yes	7.0	5.0–8.3	

FTND: Fagerstrom test for nicotine dependence; IQR = interquartile range.

^a^ Kruskal-Wallis test.

^b^ Mann-Whitney test.

^c^ Statistically significant difference between mixed-race and black race in Dunn’s post hoc test (p-value = 0.017).

Multiple linear regression analysis showed that irregular physical activity was the only factor independently associated with FTND nicotine dependence scores (β = 1.87; 95%CI: 0.55–3.19; p-value = 0.006). The adjusted model was significant (F = 5.93;p-value = 0.002), with an explanatory power of 10.0% (R^2^: 0.10) and an average VIF of 1.05. The graphical analysis showed linearity by the graph in the augmented component-plus-residual plot and normality of the residuals by the analysis of the graph and standardized normal probability plot. The model showed no specification errors (Ramsey RESET test: F = 1.20; p-value = 0.314) neither heteroscedasticity (Breusch–Pagan test *x*^2^= 0.91; p-value = 0.341).

## DISCUSSION

This investigation estimated the prevalence and factors associated with tobacco use and nicotine dependence in truck drivers in Brazil. To our knowledge, this is the first study on the determinants of the use of this substance conducted in this population in the country. The findings show a high prevalence of tobacco use and reveal its association with occupational aspects (employment contract and daily working hours). As expected, the lack of religion and use of alcohol were associated with tobacco use. Many of those that used tobacco have high/very high nicotinic dependence.

The prevalence of tobacco use in the sample was 21.1% (95%CI: 18.1–24.5), which is higher than that estimated for the general male population in Brazil (18.9%, 95%CI: 18.0–19.7)^15^, verifying the vulnerability of truck drivers to the use of this substance. In Brazil, previous studies have shown variations of 17.7%^4^ to 29.0% in the prevalence of tobacco use in truck drivers^14^. International studies in this population are also scarce; nevertheless, some investigations have found high frequencies of tobacco use in the France (51.8%), US (46.2%)^16^, and Italy (40.1%)^17^.

In this investigation, two occupational factors were associated with tobacco use: being under a permanent employment contract and daily working hours. Some studies have shown that the high workload of truck drivers, which is characterized by long daily journeys, can lead to increased fatigue, anxiety levels, depression, and occupational stress, as well as other forms of physical and psychological damage^5,16,18^. These conditions potentiate the use of licit and illicit substances by this population^18^. Regarding employment and tobacco use, we found no studies concerning the mechanisms that explain this association in the truck driver population. However, differences between truck drivers with permanent contracts and autonomous truck drivers regarding sociodemographic characteristics and risk factors for tobacco use may explain the influence of these factors. For example, a study conducted among 1,066 truck drivers in Brazil showed that permanent contract drivers have lower incomes, more compromised family lives, less flexible hours, increased work pressure, and a higher prevalence of alcohol use^13^. Another investigation conducted among 100 drivers from Campinas and São Paulo (Southeastern region) showed that drivers with permanent contracts present greater job dissatisfaction than autonomous workers^19^. Further, research conducted in 15 European countries showed that permanent workers have higher stress symptoms than other workers^20^, and this finding can be used as a proxy for comparison with the results of this study. Considering this, our finding that the abovementioned factors may contribute to increased tobacco use in truck drivers with permanent contracts when compared with that of freelancers can be said to be strongly supported. However, further research is required on the risks and mechanisms associated with the use of tobacco among truck drivers with different types of employment links.

This study verified a high prevalence of high/very high nicotine dependence among the study population. However, the absence of studies on the epidemiology of nicotine dependence prevents us from making comparisons regarding the prevalences and risk factors found in this investigation. In the general population and other specific populations, nicotinic degree and dependence are determined by a network of sociodemographic, genetic, and psychosocial factors; individual factors are represented mainly by sex, age, education, income, and marital status, whereas the most closely related occupational determinant is occupational stress^21,22^. Also, mental disorders such as depression and anxiety have a strong relationship with nicotine dependence^23^.

This study showed an association between physical inactivity and regular tobacco use/nicotine dependence, which also supports the results of other investigations^24^. Studies show that nicotine-dependent smokers are less likely to engage in regular physical activity, suffering negative health effects from smoking and indirect health effects of physical inactivity (for example: chronic non-communicable diseases)^24,26^. Some evidence suggests that, from a physiological perspective, physical activity motivates a series of neurochemical reactions that induce sensations of euphoria and pleasure, competing with or replacing the effects provoked by multiple psychoactive substances^27^. It also reduces the vulnerability to smoking, since this practice reduces anxiety and the desire to consume tobacco, especially if the physical activity is moderate to intense.

This study has some limitations. First, the cross-sectional nature of the investigation, in contrast to a longitudinal one, hinders the identification of cause-and-effect relationships between the dependent and independent’s variables investigated. Second, our non-probabilistic sample limits the generalization of the results to truck drivers from other geographic locations or to those that travel on other highways in Brazil. The sample is also not representative for all Brazilian truck drivers, since the collection took place on only one highway (BR-050); truck drivers who travel on other highways may have different sociodemographic, work, and behavioral characteristics, which may influence the magnitude of tobacco use and nicotine dependence; in addition, some drivers, depending on the employment relationship and work activities, can travel only on some highways and not others. Studies conducted with truck drivers on other highways are needed to verify these differences. Third, the data were largely self-reported, which is susceptible to memory and response bias. Fourth, the study did not use a validated instrument to measure the physical activity, anxiety, and depression and this may have caused information bias regarding these variables. Fifth, social desirability may have affected the truck drivers’ response patterns and, consequently, they may have underreported the prevalence of tobacco use and other risk factors. Six, some variables that could influence the dependent variables were not investigated, such as the influence of peers on tobacco consumption, distance traveled daily as a driver, among others. Despite these limitations, the study presents important data on the epidemiology of tobacco use and nicotine dependence in truck drivers in Brazil, which may contribute to implement control health policies and guide future research in this population.

The results of this study show a high prevalence of regular tobacco use in truck drivers who travel the BR-050 in Brazil. Occupational and behavioral determinants were associated with regular tobacco use, and high or very high nicotinic dependence was prevalent among truck drivers who smoke, being associated with irregular physical inactivity.

Health intervention planning and actions for this population should include: (I) health education campaigns in transport companies and gas stations; (II) permanent education of health professionals to implement measures to control and prevent smoking in this population, with a focus on primary health care; (III) developing strategies to facilitate access to non-medication and drug treatment for smoking and nicotine dependence in the SUS; (IV) tracking in health and worker health services of transport companies for nicotine addiction, (V) actions to expand and encourage the practice of regular physical activity in smokers and (VI) strengthening of specific and transversal policies for smoking control. These measures can contribute to reduce the magnitude of smoking and, consequently, improving the quality of life of truck drivers in Brazil. New quantitative and qualitative research is also needed to analyze the true extent of tobacco use and nicotine dependence in the truck driver population in Brazil, also analyzing new explanatory variables for these events.
